# Differential increase in prevalence estimates of inadequate sleep among black and white Americans

**DOI:** 10.1186/s12889-015-2500-0

**Published:** 2015-11-26

**Authors:** Girardin Jean-Louis, Michael A. Grandner, Shawn D. Youngstedt, Natasha J. Williams, Ferdinand Zizi, Daniel F. Sarpong, Gbenga G. Ogedegbe

**Affiliations:** Center for Healthful Behavior Change, Department of Population Health, New York University School of Medicine, 227 East 30th St, New York, NY 10016 USA; Department of Psychiatry at the University of Arizona College of Medicine, 550 East Van Buren, Tucson, AZ 85004 USA; College of Nursing and Health Innovation, College of Health Solutions, Arizona State University, Phoenix, AZ 85004 USA; Center for Minority Health & Health Disparities Research and Education, Xavier University of Louisiana, New Orleans, LA USA

**Keywords:** Inadequate sleep, Short sleep, Long sleep, Race/Ethnicity

## Abstract

**Background:**

The National Health Interview Survey (NHIS) was used to ascertain whether increases in inadequate sleep differentially affected black and white Americans. We tested the hypothesis that prevalence estimates of inadequate sleep were consistently greater among blacks, and that temporal changes have affected these two strata differentially.

**Methods:**

NHIS is an ongoing cross-sectional study of non-institutionalized US adults (≥18 years) providing socio-demographic, health risk, and medical factors. Sleep duration was coded as very short sleep [VSS] (<5 h), short sleep [SS] (5–6 h), or long sleep [LS] (>8 h), referenced to 7–8 h sleepers. Analyses adjusted for NHIS’ complex sampling design using SAS-callable SUDAAN.

**Results:**

Among whites, the prevalence of VSS increased by 53 % (1.5 % to 2.3 %) from 1977 to 2009 and the prevalence of SS increased by 32 % (19.3 % to 25.4 %); prevalence of LS decreased by 30 % (11.2 % to 7.8 %). Among blacks, the prevalence of VSS increased by 21 % (3.3 % to 4.0 %) and the prevalence of SS increased by 37 % (24.6 % to 33.7 %); prevalence of LS decreased by 42 % (16.1 % to 9.4 %). Adjusted multinomial regression analysis showed that odds of reporting inadequate sleep for whites were: VSS (OR = 1.40, 95 % CI = 1.13-1.74, *p* < 0.001), SS (OR = 1.34, 95 % CI = 1.25-1.44, *p* < 0.001), and LS (OR = 0.94, 95 % CI = 0.85-1.05, NS). For blacks, estimates were: VSS (OR = 0.83, 95 % CI = 0.60-1.40, NS), SS (OR = 1.21, 95 % CI = 1.05-1.50, *p* < 0.001), and LS (OR = 0.84, 95 % CI = 0.64-1.08, NS).

**Conclusions:**

Blacks and whites are characteristically different regarding the prevalence of inadequate sleep over the years. Temporal changes in estimates of inadequate sleep seem dependent upon individuals’ race/ethnicity.

## Background

In the last decade, the sleep literature has witnessed an important shift in examining the role of race/ethnicity in understanding determinants of habitual sleep duration at the population level [1, 2]. Evidently, race/ethnicity has been considered an important factor that was routinely adjusted in multivariate sleep models [2]. However, until recently it was not the primary focus in published sleep studies [3]. Studies with mixed results regarding whether the US population has been sleeping progressively less suggest a need for an inquiry as to whether discrepant findings may be explained by race/ethnicity, an important proxy for diverse backgrounds (e.g., socioeconomic position, culture, and health risk profile) [4].

Converging epidemiologic data have shown that individuals of the black race/ethnicity are characterized by a greater prevalence of inadequate sleep (both short and long sleep durations) [2, 5, 6]. A case in point, data from the Alameda County Health and Ways of Living Study (ACS), spanning 1965 to 1999, showed that short sleep (<7 h) was significantly more common among black respondents compared with their white counterparts [OR = 1.97; 95 % CI: 1.68-2.30] [2]. Corroborative evidence from the 2005 National Health Interview Survey demonstrated that blacks were more likely to experience short sleep (≤5 h) [12 % vs. 8 %, *p* < 0.0001] and long sleep (≥9 h) [11 % vs. 9 %, *p* < 0.0001] [5]. Similarly, data from the 1997 National Health and Nutrition Examination Survey revealed that a greater proportion of blacks (11 %), compared with whites (8 %), slept more than 8 h [6–8]. It is noteworthy that a contemporaneous report suggested that race/ethnicity was a significant predictor of inadequate sleep [OR = 1.35, 95 % CI: 1.24-1.47, *p* < 0.0001] after simultaneous adjustment for confounding effects of sociodemographic factors, depression, functional capacity, and medical illnesses [5]. In all, these findings indicate that race/ethnicity has proven an important factor in ascertaining adjusted prevalence of inadequate sleep, but the utilization of varying definitional criteria for inadequate sleep has hampered more definitive conclusions.

The National Health Interview Survey, which has monitored the health of the US population since 1957, provides a unique data set to ascertain whether increases in inadequate sleep, defined as very short (<5 h), short (5–6 h) or long (>8 h), observed at the population level differentially affect individuals of varying racial/ethnic identity. The main hypothesis tested in this inquiry is that prevalence estimates of inadequate sleep have been consistently greater among blacks, relative to whites, over the years, and that increases in prevalence estimates have affected these two strata differentially.

## Methods

The National Health Interview Survey (NHIS) is an ongoing, cross-sectional, in-person household interview survey conducted annually by the National Center for Health Statistics of the Centers for Disease Control and Prevention. The NHIS uses a multistage area probability design, sampling non-institutionalized representatives of U.S. civilian population. Probability samples of the adult population of all 50 states and the District of Columbia were obtained; the final response rate was 69 %. Surveys were conducted in both English and Spanish. Details on sample design can be found in Design and Estimation for the National Health Interview Survey, which is publicly available [9].

During face-to-face interviews conducted by trained interviewers from the US. Census Bureau, adults in the US population provided data on sociodemographic factors, health risks, and professionally diagnosed chronic conditions or diseases. Socio-demographic characteristics included age (18–85 years), sex, race/ethnicity (e.g., non-Hispanic white and non-Hispanic black), average family income dichotomized using ≥ 35K as a cut-off, and levels of education (<high school and ≥ high school including some college and college graduate). Race/ethnicity was classified according to the revised 1997 standards from the US Office of Management and Budget.

Health risk data included smoking status, alcohol intake, and physical activity. Smoking status was defined as current, former, or never. Alcohol intake was assessed based on the responses to the following questions: “In any one year, have you had at least 12 drinks of any type of alcoholic beverage?” “In your entire life, have you had at least 12 drinks of any type of alcoholic beverage?” and “In the past year, how often did you drink any type of alcoholic beverage?” Respondents who consumed <12 drinks in their entire life were classified as never-drinkers; those who consumed ≥12 drinks in any year or their entire life but not in the past year were classified as former drinkers. Current drinking was further categorized into moderate drinkers (≤7 drinks/week for women or ≤14 drinks/week for men) and heavy drinkers (>7 drinks/week for women or >14 drinks/week for men).

Physical activity was defined based on the following questions: “How often do you do vigorous leisure-time physical activities for at least 10 min that cause heavy sweating or large increases in breathing or heart rate?”, “How often do you do light or moderate leisure-time physical activities for at least 10 min that cause only light sweating or a slight to moderate increase in breathing or heart rate?”, and “How often do you do leisure-time physical activities specifically designed to strengthen your muscles such as lifting weights or doing calisthenics?” We used the recommended criterion of ≥ 150 min/week of moderate physical activity or ≥ 75 min/week of vigorous to define physical activity. If participants had indicated neither moderate nor vigorous physical activity, they were coded as having had no physical activity. However, if they had reported either moderate or vigorous or both they were classified as having engaged physical activity.

History of chronic medical diseases/conditions was captured based on affirmative responses to questions framed as follows: “Have you ever been told by a doctor or other health professional that you have [disease or condition]?” Thus, hypertension was defined by an affirmative response to: “Have you ever been told by a doctor or other health professionals that you had hypertension?” Diabetes was defined by an affirmative response to: “Other than during pregnancy, have you ever been told that you have diabetes?” Coronary heart disease was defined by an affirmative response to: “Have you ever been told that you had coronary heart disease?” Stroke was defined by an affirmative response to: “Have you ever been told that you had a stroke?” Cancer was defined by an affirmative response to: “Have you ever been told that you had cancer?”

Self-reported sleep duration was assessed with the following question: “On average, how many hours of sleep do you get in a 24-h period?” Participants estimated habitual sleep duration using full-hour increments i.e., 5 h, 6 h, 7 h, etc. with instructions to round 30 min or more up to the next whole hour and dropping 29 or fewer minutes; scores ranged from 3 to 23 h. Sleep duration was coded as either very short sleep [**VSS**] (<5 h/night), short sleep [**SS**] (5–6 h/night), or long sleep [**LS**] (>8 h/night), referenced to 7–8 h/night sleepers [10]. These cut-off points were chosen based on previous research showing health risks associated with short and long sleep durations [11, 12]. Information on both weight and height was also collected by the interviewers - this was used to generate individuals’ body mass index (BMI). Overweight was defined as BMI ≥ 25.0 and ≤ 29.9 kg/m^2^ and obesity, BMI ≥ 30 kg/m^2^, referenced to normal weight (BMI = 18.5 - 24.9 kg/m^2^).

Participants were also asked to rate their mood within the last 30 days prior to the interview. Using mood indices (e.g., feeling of sadness, hopelessness, worthlessness, and poor effort), a depression severity score was generated; this was a composite score estimated using the K-6 scaling system [13]. Responses were used to generate a score ranging from 0 to 24. Scores ≥ 13 indicated a greater degree of depression - this group was defined as having emotional distress [14]. The National Health Interview Survey is subject to the Ethics Review Board (ERB) of the Center for Disease Control and Prevention and the National Center for Health Statistics. The data are publicly available.

### Statistical analysis

Analyses were based on NHIS data obtained from 1977 through 2009. Since the NHIS dataset includes data from different samples using a multistage area probability sampling design, all analyses performed in this study were based on weighted statistics using the final weights provided with the NHIS dataset. These weights represent a product of weights for corresponding units computed in each of the sampling stages. Once these data were harmonized and standardized across available years, additional processing was unnecessary. As the Center for Disease Control and Prevention recommends, the complex survey analysis techniques we used accounted for the weights, strata and clusters in the NHIS design.

An analytic sample (with varied sample size by year) was obtained by excluding data using the following criteria: non-whites, non-blacks or missing data for race; data on all key variables classifies as not in universe (NIU), missing, refused, or don’t know/unknown; age < 18 years or age > 70 years; BMI < 15 kg/m^2^ or BMI > 70 kg/m^2^; hours of sleep < 2 or hours of sleep > 20; and marital status classified as NIU or unknown. Since the study hypothesis focused on black and white respondents, data from other race/ethnic strata were excluded. Sample characteristics were described using measures of central tendency and dispersion or using frequency distributions based on the nature of the data (continuous versus categorical). All analyses took into account the complex sampling design of NHIS and were performed using SAS version 9.1.2 (SAS Institute, Cary, N.C.) and SAS-callable SUDAAN version 9 (RTI International, Research Triangle Park, N.C.) [15, 16].

First, preliminary analysis aggregating 1977–2009 data was conducted, showing significant interactions between sleep duration and race/ethnicity for many of the factors of interest. Thus, age and sex-adjusted descriptive analysis of the prevalence of inadequate sleep was performed separately for each stratum across all available years of observation (Tables 1 and 2); descriptive characteristics are also indicated. Next, to ascertain factors (see below) likely to be associated with inadequate sleep within each stratum, multinomial logistic regression modeling for complex survey (Proc Survey logistic; SAS 9.1.2) was used, yielding age and sex-adjusted prevalence of inadequate sleep (Tables 3 and 4). Lastly, multinomial logistic regression modeling was also applied to assess changes in prevalence estimates from 1983 to 2009 in both strata, adjusting effects of all sociodemographics, health risks, and medical conditions.

**Table 1:** Characteristics of white participants in the NHIS study (1977-2009)

Characteristics	1977	1983	1985	1990	2004	2005	2006	2007	2008	2009
N	16,429	8,576	23,756	29,028	19,357	19,510	14,089	13,720	12,814	16,281
Gender, Female (%)	54.2	55.8	51.3	51.2	49.9	49.9	49.5	49.3	49.5	49.8
Age, years (mean ± SD)	42 ± 15	40 ± 15	40 ± 17	41 ± 17	42 ± 17	42 ± 17	42 ± 17	43 ± 18	43 ± 18	43 ± 19
Education, ≥ HS (%)	73.0	78.6	81.2	83.5	88.1	88.3	87.7	88.7	88.5	88.8
Marital status (%)										
Married	74.5	69.6	69.5	69.6	62.1	61.8	60.9	59.9	59.3	57.9
Widowed, Divorced & Separated	12.3	13.0	11.7	12.6	15.2	15.3	15.7	15.5	16.1	16.4
Single	13.2	17.4	18.8	17.8	22.7	22.9	23.4	24.6	24.6	25.7
Total family income, ≥ 35K (%)	--	23.7	32.0	48.3	66.6	67.8	67.9	71.5	72.9	72.5
Poverty status, below (%)	--	6.6	8.8	7.3	9.1	9.0	10.2	9.5	10.3	10.8
Ever smoked, yes (%)	58.8	56.3	56.5	52.5	44.2	44.3	44.0	43.7	44.2	44.6
Alcohol consumption										
Never drinkers	--	--	--	13.0	19.6	19.0	19.6	18.3	16.3	15.3
Former drinkers	--	--	--	9.0	13.2	12.8	12.9	13.1	12.4	13.1
Current drinkers	--	--	--	78.0	67.2	68.2	67.5	68.6	71.2	71.5
Body mass index (mean ± SD)	24.3 ± 4.3	24.4 ± 4.5	24.6 ± 5.1	25.0 ± 5.1	26.6 ± 5.6	26.8 ± 5.7	27.1 ± 6.4	27.0 ± 6.3	27.3 ± 6.4	27.3 ± 6.8
Obesity (BMI ≥ 30 kg/m^2^)	9.1	10.3	10.8	12.9	22.9	23.9	25.7	25.5	26.8	26.8
Physical activity, yes (%)	--	--	--	--	10.9	11.4	11.2	11.3	11.5	12.5
Emotional stress, yes (%)	--	--	--	--	3.3	3.0	3.1	2.7	3.6	3.5
Comorbid conditions:										
Diabetes	--	3.4	--	--	5.9	6.5	6.6	6.2	7.1	7.9
Hypertension	--	20.7	21.4	19.9	20.3	20.4	22.3	22.0	23.9	23.4
Cancer	--	3.2	--	--	5.5	6.1	5.8	6.1	6.6	6.8
Coronary heart disease	--	1.8	--	--	2.7	3.1	2.9	2.9	2.7	3.2
Heart attack (MI)	--	--	--	--	2.2	2.4	2.3	2.1	2.5	2.3
Stroke	--	0.9	1.2	1.2	1.5	1.2	1.4	1.5	1.8	1.6
Kidney disease	--	--	--	--	1.2	1.2	1.1	1.0	1.2	1.4
Sleep measures:										
Hours of sleep (mean ± SD)	7.4 ± 1.2	7.3 ± 1.2	7.4 ± 1.4	7.3 ± 1.4	7.1 ± 1.4	7.1 ± 1.5	7.1 ± 1.3	7.1 ± 1.4	7.1 ± 1.5	7.1 ± 1.7
Categories of sleep quantity										
Very short sleep	1.5	1.7	1.1	1.4	2.0	2.1	2.1	1.8	2.3	2.3
Short sleep	19.3	20.1	20.2	22.3	26.0	26.0	26.2	24.7	25.6	25.4
Normal sleep	68.0	69.0	68.7	68.2	64.9	64.9	64.9	66.9	64.9	64.5
Long sleep	11.2	9.1	10.0	8.1	7.0	7.0	6.8	6.6	7.2	7.8

Legend: Note that several health factors were not collected prior to 2004

**Table 2:** Characteristics of black participants in the NHIS study (1977-2009)

Characteristics	1977	1983	1985	1990	2004	2005	2006	2007	2008	2009
N	1,703	942^a^	4,270	4,922	3,501	3,496	3,285	2,977	2,727	3,738
Gender, Female (%)	59.1	59.5	55.3	54.8	54.2	53.1	53.6	53.2	54.5	54.5
Age, years (mean ± SD)	40 ± 15	38 ± 15	38 ± 19	38 ± 18	40 ± 17	40 ± 17	40 ± 18	40 ± 17	40 ± 18	41 ± 16
Education, ≥ HS (%)	55.2	64.9	67.0	72.3	84.5	85.2	86.2	86.4	87.3	86.9
Marital status (%)										
Married	50.3	47.0	46.7	45.9	38.9	37.7	37.9	37.4	35.3	36.0
Widowed, Divorced & Separated	29.3	21.8	20.6	21.5	22.4	22.0	21.2	22.2	20.6	21.0
Single	20.4	31.2	32.7	32.6	38.7	40.3	40.9	40.4	44.1	43.0
Family income, ≥35K (%)	--	10.3	13.5	25.4	45.9	47.2	45.3	52.3	54.9	53.0
Poverty status, below (%)	--	22.8	27.9	23.5	18.5	20.4	21.6	23.1	19.9	23.0
Ever smoked, yes (%)	57.2	50.5	52.1	43.5	32.8	34.7	34.3	33.3	34.9	35.2
Alcohol consumption										
Never drinkers	--	--	--	25.4	36.2	36.4	34.1	31.9	28.9	27.1
Former drinkers	--	--	--	9.3	13.2	13.0	13.8	14.5	14.9	15.1
Current drinkers	--	--	--	65.4	50.6	50.6	52.2	53.6	56.2	57.8
Body mass index (mean ± SD)	25.8 ± 5.4	25.6 ± 5.2	25.6 ± 6.2	26.5 ± 6.4	28.0 ± 6.3	27.8 ± 6.3	28.3 ± 7.2	28.4 ± 7.1	28.4 ± 7.3	28.7 ± 8.2
Obesity (BMI ≥ 30 kg/m^2^)	16.9	17.6	16.1	21.3	31.9	31.3	32.9	34.4	35.9	36.7
Physical activity, yes (%)	--	--	--	--	11.5	11.5	10.9	11.9	13.6	13.7
Emotional stress, yes (%)	--	--	--	--	3.6	3.2	3.7	2.6	3.6	4.0
Comorbid conditions:										
Diabetes	--	5.8	--	--	8.3	8.7	9.7	9.5	9.4	10.7
Hypertension	--	31.2	28.1	28.0	27.8	27.5	28.9	29.9	30.6	30.9
Cancer	--	0.3	--	--	2.7	2.4	2.8	2.9	2.6	3.2
Coronary heart disease	--	1.4	--	--	2.2	2.5	2.5	2.6	2.3	2.9
Heart attack (MI)	--	--	--	--	1.4	1.6	2.1	1.6	2.3	2.5
Stroke	--	1.1	1.3	1.8	2.1	2.0	2.7	2.4	2.5	2.4
Kidney disease	--	--	--	--	1.8	1.9	1.5	2.6	1.6	1.6
Sleep measures:										
Hours of sleep (mean ± SD)	7.4 ± 1.6	7.2 ± 1.6	7.5 ± 2.3	7.4 ± 2.4	7.1 ± 2.3	7.1 ± 1.7	7.0 ± 1.8	7.1 ± 1.8	7.1 ± 2.1	7.0 ± 2.3
Categories of sleep quantity										
Very short sleep	3.3	4.4	2.4	2.4	3.3	3.1	3.7	3.6	3.8	4.0
Short sleep	24.6	27.8	25.8	27.4	31.7	31.7	32.6	30.9	33.2	33.7
Normal sleep	56.0	55.9	54.6	56.2	55.5	55.7	55.4	56.5	53.9	52.9
Long sleep	16.1	11.9	17.2	14.0	9.4	9.4	8.3	9.0	9.1	9.4

Legend: Note that several health factors were not collected prior to 2004; ^a^the total sample is 1,934, but only 942 were usable in analysis due to application of weighting factors

**Table 3:** Age-sex-adjusted associations of inadequate sleep with sociodemographics, health risks, and comorbid conditions in the white stratum (1983-2009)

Characteristics	Very short sleep	Short sleep	Long sleep
	OR (95 % CI)	OR (95 % CI)	OR (95 % CI)
Education (*Reference:* ≥ *High School*)	2.04 (1.83,2.28)^b^	1.06 (1.01,1.10)^b^	1.89 (1.79,2.01)^b^
Marital status (*Reference: Married*)			
Widowed	2.31 (1.92,2.77)^b^	1.43 (1.32,1.53)^b^	1.51 (1.35,1.69)^b^
Divorced	2.72 (2.41,3.07)^b^	1.58 (1.51,1.65)^b^	1.19 (1.11,1.28)^b^
Separated	3.90 (3.20,4.76)^b^	1.64 (1.50,1.78)^b^	1.29 (1.21,1.49)^b^
Single	1.35 (1.17,1.56)^b^	0.96 (0.92,1.00)	1.68 (1.59,1.79)^b^
Total family income (Reference: ≥ 35K)	2.08 (1.89,2.30)^b^	1.06 (1.03,1.09)^b^	1.79 (1.69,1.88)^b^
Poverty status (*Reference: Above*)	2.95 (2.60,3.34)^b^	1.20 (1.14,1.26)^b^	1.94 (1.80,2.08)^b^
Smoking status (*Reference: Never*)	1.06 (1.05,1.07)^b^	1.03 (1.03,1.03)^b^	1.04 (1.03,1.04)^b^
Alcohol consumption (*Reference: Never*)			
Former	2.12 (1.74,2.57)^b^	1.59 (1.49,1.69)^b^	1.37 (1.24,1.51)^b^
Current	1.00 (0.87,1.15)	1.30 (1.24,1.36)^b^	0.80 (0.75,0.86)^b^
Physical activity (Reference: Yes)^a^	4.35 (3.48,5.44)^b^	1.52 (1.33,1.75)^b^	3.12 (2.61,3.75)^b^
Emotional distress (Reference: No)^a^	9.73 (7.18,13.17)^b^	2.46 (1.98,3.07)^b^	3.17 (2.34,4.25)^b^
Body mass index (*Reference: Normal*)			
Overweight	1.23 (1.10,1.39)^b^	1.18 (1.14,1.22)^b^	0.98 (0.93,1.04)
Obesity	2.12 (1.88,2.39)^b^	1.57 (1.51,1.64)^b^	1.19 (1.12,1.27)^b^
Comorbid conditions:			
Cancer	1.69 (1.42,2.02)^b^	1.13 (1.05,1.21)^b^	1.50 (1.34,1.68)^b^
Diabetes	2.22 (1.88,2.63)^b^	1.23 (1.15,1.32)^b^	1.97 (1.77,2.18)^b^
Hypertension	2.11 (1.90,2.36)^b^	1.28 (1.23,1.32)^b^	1.50 (1.42,1.59)^b^
Kidney disease	3.27 (2.40,4.47)^b^	0.58 (0.49,0.67)^b^	1.81 (1.47,2.24)^b^
Heart attack (MI)	3.05 (2.30,3.88)^b^	1.26 (1.12,1.42)^b^	2.58 (2.19,3.04)^b^
Coronary heart disease	2.34 (1.88,2.91)^b^	1.16 (1.05,1.29)^b^	2.24 (1.93,2.61)^b^
Stroke	4.47 (3.56,5.63)^b^	1.47 (1.31,1.66)^b^	3.26 (2.81,3.78)^b^

Legend: ^a^Data available for 2004 – 2009; ^b^denotes significance at the 5 % level (i.e., odds ratio is either significantly < or > 1); data from 1977 were excluded since many of the medical comorbidities were not available; inadequate sleep was defined as very short sleep [VSS] (<5 h/night), short sleep [SS] (5-6 h/night), or long sleep [LS] (>8 h/night), referenced to 7-8 h/night sleepers

**Table 4:** Age-sex-adjusted associations of inadequate sleep with sociodemographics, health risks, and comorbid conditions in the black stratum (1983-2009)

Characteristics	Very short sleep	Short sleep	Long sleep
	OR (95 % CI)	OR (95 % CI)	OR (95 % CI)
Education (*Reference:* ≥ *High School*)	1.22 (1.01,1.47)^b^	0.82 (0.75,0.89)^b^	2.12 (1.90,2.36)^b^
Marital status (*Reference: Married*)			
Widowed	1.12 (0.77,1.63)	1.01 (0.86,1.19)	1.43 (1.16,1.77)^b^
Divorced	1.61 (1.25,2.06)^b^	1.10 (1.00,1.21)^b^	0.89 (0.76,1.04)
Separated	1.54 (1.14,2.09)^b^	1.02 (0.89,1.16)	1.32 (1.10,1.60)^b^
Single	1.15 (0.91,1.45)	0.88 (0.81,0.96)^b^	1.19 (1.06,1.34)^b^
Family income (Reference: ≥ 35K)	1.52 (1.25,1.85)^b^	0.81 (0.75.0.87)^b^	1.77 (1.56,1.99)^b^
Poverty status (*Reference: Above*)	1.95 (1.61,2.36)^b^	0.96 (0.88,1.04)	1.94 (1.73,2.17)^b^
Smoking status (*Reference: Never*)	1.04 (1.02,1.06)^b^	1.02 (1.01,1.02)^b^	1.05 (1.04,1.06)^b^
Alcohol consumption (*Reference: Never*)			
Former	1.67 (1.26,2.20)^b^	1.51 (1.34,1.70)^b^	1.29 (1.08,1.54)^b^
Current	1.52 (1.22,1.89)^b^	1.58 (1.45,1.72)^b^	1.02 (0.90,1.16)
Physical activity (Reference: Yes)^a^	2.51 (1.62,3.90)^b^	1.02 (0.78,1.35)	2.90 (2.07,4.05)^b^
Emotional distress (Reference: No)^a^	13.95 (11.89,16.37)^b^	2.70 (2.44,2.99)^b^	4.08 (3.53,4.71)^b^
Body mass index (*Reference: Normal*)			
Overweight	1.38 (1.11,1.70)^b^	1.17 (1.08,1.27)^b^	0.87 (0.77,0.98)
Obesity	1.73 (1.39,2.15)^b^	1.36 (1.25,1.48)^b^	1.08 (0.92,1.18)
Comorbid conditions:			
Cancer	1.27 (1.04,1.94)^b^	1.24 (1.01,1.53)^b^	1.36 (0.99,1.88)
Diabetes	1.50 (1.05,2.15)^b^	1.13 (1.04,1.29)^b^	1.46 (1.21,1.76)^b^
Hypertension	1.69 (1.39,2.05)^b^	1.09 (1.01,1.18)^b^	1.32 (1.18,1.49)^b^
Kidney disease	2.27 (1.38,3.74)^b^	0.86 (0.65,1.13)	1.49 (1.02,2.31)^b^
Heart attack (MI)	1.71 (1.05,2.79)^b^	1.08 (0.83,1.39)	2.23 (1.56,3.18)^b^
Coronary heart disease	1.83 (1.15,2.92)^b^	1.09 (0.87,1.37)	1.61 (1.32,2.35)^b^
Stroke	2.35 (1.57,3.51)^b^	0.86 (0.69,1.08)	1.78 (1.37,2.37)^b^

Legend: ^a^Data available for 2004 – 2009; ^b^denotes significance at the 5 % level (i.e., odds ratio is either significantly < or > 1); data from 1977 were excluded since many of the medical comorbidities were not available; inadequate sleep was defined as very short sleep [VSS] (<5 h/night), short sleep [SS] (5-6 h/night), or long sleep [LS] (>8 h/night), referenced to 7-8 h/night sleepers

Associations of several sociodemographics (education, income, marital status, poverty), health risks (smoking status, alcohol consumption, physical activity, and emotional distress), and medical conditions (hypertension, diabetes, stroke, heart attack and heart disease) with inadequate sleep were ascertained. These factors were chosen because preliminary analyses demonstrated that many of these factors changed over time (Tables 1 and 2), with potential adverse effects on inadequate sleep [1, 2]. A review of all factors likely to influence sleep duration has been recently published [17].

## Results

### Characteristics of the NHIS sample

Characteristics of the sample, including sociodemographics, health risks, and medical conditions are reported in Tables 1 and 2. Data for each year of NHIS data capture are presented separately for whites and blacks. Among whites, the prevalence of VSS increased by 53 % (1.5 % to 2.3 %) from 1977 to 2009 and the prevalence of SS increased by 32 % (19.3 % to 25.4 %); prevalence of LS decreased by 30 % (11.2 % to 7.8 %). Among blacks, the prevalence of VSS increased by 21 % (3.3 % to 4.0 %) and the prevalence of SS increased by 37 % (24.6 % to 33.7 %); prevalence of LS decreased by 42 % (16.1 % to 9.4 %). Notably, differences between 2004 and 2009 were generally smaller, with a 15 % increase in VSS, a 2 % decrease in SS, and an 11 % increase in LS among whites, and a 21 % increase in VSS, a 6 % increase in SS, and no change in LS among blacks. Of interest was the observation that prevalence estimates for blacks and whites reporting sleeping 7–8 h decreased from 1977 to 2009 by 6 % (56.0 % to 52.9) and 5 % (68.0 % to 64.5 %), respectively.

Preliminary analysis aggregating 1977–2009 data was conducted, indicating that overall blacks had 58 % greater odds of being insufficient sleepers [short or very short sleep; < 7 h] (OR = 1.58, 95 % CI = 1.52-1.63, *p* < 0.001) and a 62 % greater odds of being long sleepers (OR = 1.62, 95 % CI = 1.54-1.71, *p* <0.001) compared with whites. They also showed significant interactions between sleep duration and race/ethnicity for many of the factors of interest (Tables 1 and 2); for instance, black insufficient or long sleepers were 77 % (OR = 1.77, 95 % CI = 1.29, 2.44, *p* < 0.001) and 63 % (OR = 1.63, 95 % CI = 1.35-1.97, *p* < 0.001) more likely to be obese, relative to whites.

### Associations of sociodemographics, health risks, and medical conditions with inadequate sleep

Analyses depicting associations of sociodemographics, health risks, and medical conditions with inadequate sleep are presented in Tables 3 and 4. Accordingly, among whites, lower education level, being widowed, divorced, or separated, having lower income, being poor, smoking, being a former drinker, physical inactivity, emotional distress, a history of cancer, diabetes, hypertension, heart attack, coronary heart disease, and stroke were all associated with increased likelihood of VSS and SS; being single was associated with VSS only and being a current drinker was only associated with SS. History of kidney disease was associated with increased likelihood of VSS, but was associated with decreased likelihood of SS. Regarding LS, lower education, non-married status, smoking, being a former drinker, physical inactivity, emotional distress, and all medical comorbidities were associated with increased likelihood of LS; current drinking was associated with decreased likelihood of LS.

Among blacks, lower education, non-married status, lower income, being poor, smoking history, drinking history (current or former), physical inactivity, emotional distress, and all medical comorbidities were associated with increased likelihood of VSS. History of drinking (current or former), emotional distress, history of cancer, diabetes, or hypertension were associated with increased likelihood of SS; lower education, single status, and lower income were associated with decreased likelihood. Regarding LS, lower education, being widowed, separated, or single, lower income, being poor, smoking history, being a former drinker, physical inactivity, emotional distress, history of diabetes, hypertension, kidney disease, heart attack, coronary heart disease, and stroke were associated with increased likelihood of reporting LS.

Adjusted multinomial regression analysis, ascertaining changes in prevalence estimates from 1983 to 2009, showed that odds of reporting inadequate sleep for whites were as follows: VSS (OR = 1.40, 95 % CI = 1.13-1.74, *p* < 0.001), SS (OR = 1.34, 95 % CI = 1.25-1.44, *p* < 0.001), and LS (OR = 0.94, 95 % CI = 0.85-1.05, NS). For blacks, estimates were: VSS (OR = 0.83, 95 % CI = 0.60-1.40, NS), SS (OR = 1.21, 95 % CI = 1.05-1.50, *p* < 0.001), and LS (OR = 0.84, 95 % CI = 0.64-1.08, NS). In both models, effects of sociodemographics, health risks, and medical conditions were adjusted.

## Discussion

Consistent with the mandate of the Institute of Medicine for increased surveillance of inadequate sleep in the US population [18], many epidemiologic studies have been conducted to document both the prevalence and severity of this public health burden [7, 19–24]. These studies have engendered an important debate as to whether the population’s overall sleep time has declined [4, 20, 25]. While our investigation did not aim to settle the debate one way or the other, it is in tandem with evidence indicating that individuals’ race/ethnicity significantly influences the prevalence of reported inadequate sleep (sleeping too little or too much) [26–30]. Our investigation expands on the extant epidemiologic sleep literature demonstrating that changes in prevalence estimates in the last 3 decades may be in part dependent upon individuals’ race/ethnicity.

Previously, we reported results of our analysis of NHIS data supporting the notion that the prevalence of insufficient sleep in the US population has increased over last three decades [31]. These findings along with other analyses of epidemiologic sleep data had suggested a gradual decline in habitual sleep time with important ramifications vis-a-vis elevated risks of chronic diseases [32–34], early mortality [35, 36], decrements in neurobehavioral and job performance [37–39], decreased quality of life, and increased healthcare costs [40, 41]. The decrease in modal sleep duration (1.5 h) from 1959 to 2005 might have been assumed to affect all strata of the US population equally [4, 22]. Results of our prevalence estimates challenge this assumption and expand on the literature in two meaningful ways.

First, they demonstrate that the prevalence of insufficient sleep may be recalcitrantly different for various strata of the US population. Our analyses indicated that from 1977 to 2009 prevalence estimates of VSS and SS among blacks have been consistently greater than those of their white counterparts (Tables 1 and 2). These results are consistent with a previous report that also evidenced insufficient sleep (<7 h) was more prevalent among blacks [OR = 1.97; 95 % CI = 1.68-2.30] [2, 5]. However, since we utilized a more nuanced categorization scheme for defining insufficient sleep (VSS and SS), we were able to perform a more detailed examination of degrees of insufficient sleep that blacks and whites experienced habitually. In effect, overall the prevalence of both VSS and SS was substantially greater among blacks. Hence, black very-short sleepers may be at greater risk of experiencing deleterious physiologic and hormonal effects of insufficient sleep, which may predispose them to adverse cardiometabolic outcomes, as has been demonstrated in experimental sleep curtailment studies [34, 42–45].

Second, our findings make a significant contribution inasmuch as they reveal that gradual increases in prevalence estimates of VSS and SS in the last three decades appear to have affected blacks and whites differentially. Results showed that from 1977 to 2009 prevalence estimates of VSS and SS within the white stratum increased by 53 % and 32 %, respectively. Prevalence estimates of VSS and SS within the black stratum also increased, but varied considerably from what might have been expected; estimates of VSS and SS were 21 % and 37 %, respectively. Thus, increases in the prevalence of VSS seem to be relatively greater among whites, whereas increases in the prevalence of SS appear to be greater among blacks. We surmise that population-based interventions aiming at increasing sleep duration should be tailored to address specific factors underlying varying prevalence estimates. Conceivably, factors perpetuating short sleep among blacks should be the appropriate focus of intervention, whereas those spurring gradual increases in very short sleep among whites should be the focus.

We were not able to discern which specific factor set might explain disparate increases in prevalence estimates from 1977 to 2009, but racial/ethnic differences in demographics, health risks, and medical conditions seem to have played an important role. Indeed, after adjusting effects of these factors in the logistic models, prevalence estimates of VSS and SS in these two strata were generally much closer. Plausibly, discrepancies reported in the literature regarding increases in the prevalence of insufficient sleep (VSS and SS) may be in part explained by lack of detailed within-group analysis likely to reveal divergent prevalence estimates, as demonstrated in our analyses. Of interest in this regard are other studies that have also shown the importance of considering individuals’ race-ethnicity in analysis of sleep data. In sum, they have led to the belief that factors likely to influence habitual sleep time are generally more common among blacks. For instance, socioeconomic position, which is historically lower among blacks, is a prominent determinant of insufficient sleep. Indeed, a previous study revealed that socioeconomic position remained a significant predictor of insufficient sleep even after adequate adjustment for health-related characteristics linked to insufficient sleep. Consistent with this previous study, prevalence estimates of insufficient sleep in our investigation remained considerably high after adjusting effects of poverty status, which is often more deterministic than family income, employment status, or type of occupation [46]. Other factors that may differentially affect insufficient sleep among blacks include urban residence [47], work schedule [48, 49], job strain [50], lack of emotional support [51], perceived discrimination [52, 53], types of industry and occupation [17, 46, 54], and high burden of chronic medical conditions [55]. Thus, investigations of potential increases in the prevalence of insufficient sleep at the population level should incorporate comprehensive subgroup analyses to avoid imprecise epidemiologic estimates.

When viewed in the context of health equity research, insufficient sleep may be a key factor in understanding diseases that disproportionately burden blacks. Whether sleep time in this population is curtailed by lifestyle choices or restricted by sleep fragmentation as is the case in sleep apnea, blacks may be at increased risk of developing chronic diseases (e.g., diabetes and cardiovascular disease) due to insufficient sleep. Of note, the relatively high prevalence of insufficient sleep could be partially attributed to untreated sleep apnea. An important study comparing 225 black and 622 white volunteers, ages 2–86 years, showed that 31 % of blacks versus 10 % of whites had sleep apnea [56]. Furthermore, data from the Nurse’s Health Study suggested that insufficient sleep resulting from sleep apnea may lead to the development or exacerbation of diabetes [57, 58]. It has been shown that insufficient sleep is a precursor of inflammatory processes, which are important risk markers for diabetes and cardiovascular disease [59–61]. Unfortunately, the NHIS database does not include data permitting delineation of the effects of such factors. Notwithstanding this limitation, it is noteworthy that analysis of other sleep-related factors has shown that they do not uniformly affect inadequate sleep of black and white respondents, although the medical factors appear to have exhibited the most commonality (Tables 3 and 4). Hence, interventions aiming at addressing inadequate sleep in these two strata should consider which characteristics are the best modifiable risk factors to incorporate in tailored sleep health interventions.

Another important observation in our study was that differences observed in the prevalence of insufficient sleep from 2004 to 2009 were generally smaller for both strata, leading to the notion that the prevalence of insufficient sleep may have reached a plateau. This observation is important, but has not been fully elucidated although it is in line with observations made in two previous analyses. While the first one surveying US adults between 1985 and 2006 revealed a general increase in the prevalence of insufficient sleep [62], the second one, investigating a similar sample of US adults between 2004 and 2007, showed a decrease in prevalence estimates [17]. It is of interest to examine whether observations made in our analyses of NHIS data could be replicated in other epidemiologic sleep data collected over the same time span and, perhaps more importantly, whether differences noted in these two racial/ethnic strata could be replicated. In sum, our results are consistent with analysis relying on long periods of observations, showing consistent and gradual increases in the prevalence of insufficient sleep [2, 63].

The observation that blacks were characterized by a greater prevalence of LS than were whites is consistent with previous reports. One such report was based on analysis of the 2005 National Health Interview Survey, which demonstrated that blacks were more likely to experience long sleep (≥9 h) [11 % vs. 9 %, *p* < 0.0001] [5]. Another report based on analysis of the 1997 National Health and Nutrition Examination Survey indicated that a greater proportion of blacks (11 %), compared with whites (8 %), slept more than 8 h [6]. Our findings expand on previous observations regarding LS, evidencing that both white and blacks exhibited decreases in the prevalence of LS from 1977 to 2009, albeit to varying degrees (whites = 30 % and blacks = 42 %). These findings seem commensurate with relative increases in the prevalence of both VSS and SS. Taken together, these findings suggest that the categorization scheme used in our analyses, rather than the average sleep duration, may be a superior index to assess potential differences in epidemiologic sleep profile of blacks and whites.

The observation that prevalence estimates for blacks and whites reporting sleeping 7–8 h decreased by 6 % and 5 %, respectively from 1977 to 2009 is important. This implies that although both strata have exhibited substantial variations regarding the prevalence of inadequate sleep, they were remarkably similar insofar as decreases in the number of 7–8 h sleepers is concerned. Furthermore, the number of respondents reporting sleeping 7–8 h has not changed as drastically as we observed for those reporting inadequate sleep. This is seemingly a function of the fact that although the number of insufficient sleepers has increased generally, there has been a commensurate decrease in the number of long sleepers, the latter of which could be associated with positive health outcomes.

## Conclusions

Future studies should investigate the mediators of greater prevalence of inadequate sleep observed in the black stratum, relative to the white stratum. Our analyses adjusted effects of 17 covariates, which have shown significant associations in previous research. They should also examine the accuracy of self-reported sleep time, particularly in the black racial/ethnic stratum. Evidence from appraisal research suggests that recall bias may influence the accuracy of self-reported data among blacks [64–68]. An important limitation of the current findings is that both sleep duration and medical data were based on subjective reports obtained by a trained interviewer. It is not certain what the true prevalence of insufficient sleep might be as evidence suggests that insufficient sleepers tend to overestimate their sleep time, or the true prevalence of long sleep given that these individuals may spend a long time in bed while not sleeping [66, 69–72]. It is of interest to examine whether inadequate sleep measured with actigraphy would support self-reported data indicating differences between blacks and whites. While there has not been a large representative population-based actigraphic study, community-based actigraphic data have shown differences in inadequate sleep mirroring the ones observed in the present investigation [22]. On balance, we should also note that objective sleep measures have not yielded consistent evidence that average sleep time at the population level has declined [73]. Despite the inherent limitation of the data, one of the important strengths of this investigation relates to the application of robust analytic techniques to a single large dataset, evidencing that indeed blacks and whites are characteristically different with regard to the prevalence of inadequate sleep over the years, which may explain disparities in cardiometabolic risks [74]. Lastly, results also indicate that temporal changes in estimates of inadequate sleep seem dependent upon individuals’ racial/ethnic background.

## References

[1] Stranges S, Dorn JM, Shipley MJ, Kandala NB, Trevisan M, Miller MA (2008). Correlates of short and long sleep duration: a cross-cultural comparison between the United Kingdom and the United States: the Whitehall II Study and the Western New York Health Study. Am J Epidemiol.

[2] Stamatakis KA, Kaplan GA, Roberts RE (2007). Short sleep duration across income, education, and race/ethnic groups: population prevalence and growing disparities during 34 years of follow-up. Ann Epidemiol.

[3] Leger D, Guilleminault C, Defrance R, Domont A, Paillard M (1999). Prevalence of sleep/wake disorders in persons with blindness. Clin Sci (Colch).

[4] Bin YS, Marshall NS, Glozier N (2012). Secular trends in adult sleep duration: a systematic review. Sleep Med Rev.

[5] Nunes J, Jean-Louis G, Zizi F, Casimir GJ, von Gizycki H, Brown CD (2008). Sleep duration among black and white Americans: results of the National Health Interview Survey. J Natl Med Assoc.

[6] Qureshi AI, Giles WH, Croft JB, Bliwise DL (1997). Habitual sleep patterns and risk for stroke and coronary heart disease: a 10-year follow-up from NHANES I. Neurology.

[7] Gallup: Omnibus Sleep in America Poll. The Gallup Organization 1998, 1–70. https://sleepfoundation.org/; accessed 12,1998.

[8] Gallup. 2000 Omnibus Sleep in America Poll. National Sleep Foundation, 1–19. 2000. National Sleep Foundation. https://sleepfoundation.org/; accessed 9,2000.

[9] Botman SL, Moore TF, Moriarity CL, Parsons VL: Design and estimation for the National Health Interview Survey, 1995–2004. National Center for Health Statistics. Vital Health Stat. 2000;130:1–31. 11707926

[10] National Institutes of Health. Your guide to healthy sleep. 2012. http://catalog.nhlbi.nih.gov/catalog/product/Your-Guide-to-Healthy-Sleep/11-5271; accessed 9,2014.

[11] Kripke DF, Garfinkel L, Wingard DL, Klauber MR, Marler MR. Mortality associated with sleep duration and insomnia. Arch Gen Psychiatry. 2002;59:131–6. 10.1001/archpsyc.59.2.131 11825133

[12] Tamakoshi A, Ohno Y (2004). Self-reported sleep duration as a predictor of all-cause mortality: results from the JACC study, Japan. Sleep.

[13] Kessler RC, Barker PR, Colpe LJ, Epstein JF, Gfroerer JC, Hiripi E (2003). Screening for serious mental illness in the general population. Arch Gen Psychiatry.

[14] Kessler RC, Green JG, Gruber MJ, Sampson NA, Bromet E, Cuitan M (2010). Screening for serious mental illness in the general population with the K6 screening scale: results from the WHO World Mental Health (WMH) survey initiative. Int J Methods Psychiatr Res.

[15] Research Triangle Institute (2004). SUDAAN user’s manual, Release 9.0.

[16] SAS Institute Inc (2008). SAS/STAT® 9.2. User’s Guide.

[17] Luckhaupt SE, Tak S, Calvert GM (2010). The prevalence of short sleep duration by industry and occupation in the National Health Interview Survey. Sleep.

[18] IOM Report on Sleep Disorders and Sleep Deprivation: An Unmet Public Health Problem. IOM. 2006. http://www.ncbi.nlm.nih.gov/books/NBK19960/; accessed 6,2014. 20669438

[19] Gallup: The Gallup Study of Sleeping Habits. The Gallup Organization. Princeton NJ. 1979, 1–60.

[20] Schoenborn CA (1986). Health habits of U.S. adults, 1985: the “Alameda 7” revisited. Public Health Rep.

[21] Gallup: Sleep In America. The Gallup Organization. Princeton NJ. 1995, 1–78. https://sleepfoundation.org/; accessed 6,1996.

[22] Jean-Louis G, Kripke DF, Ancoli-Israel S, Klauber M, Sepulveda RS (2000). Sleep duration, illumination, and activity patterns in a population sample: Effects of gender and ethnicity. Biol Psychiatry.

[23] National Sleep Foundation. Sleep in America Poll, Washington, DC. National Sleep Foundation. 1–51. 2005. National Sleep Foundation. https://sleepfoundation.org/; accessed 11-2007.

[24] The National Institutes of Diabetes & Digestive & Kidney Diseases. National Institutes of Health. http://www.nih.gov/about-nih/what-we-do/nih-almanac/national-institute-diabetes-digestive-kidney-diseases-niddk; Accessed 11-20-2009.

[25] Flegal KM, Carroll MD, Ogden CL, Curtin LR (2010). Prevalence and trends in obesity among US adults, 1999–2008. JAMA.

[26] Singh M, Drake CL, Roehrs T, Hudgel DW, Roth T (2005). The association between obesity and short sleep duration: a population-based study. J Clin Sleep Med.

[27] Lopez-Garcia E, Faubel R, Leon-Munoz L, Zuluaga MC, Banegas JR, Rodriguez-Artalejo F (2008). Sleep duration, general and abdominal obesity, and weight change among the older adult population of Spain. Am J Clin Nutr.

[28] Patel SR, Hu FB (2008). Short sleep duration and weight gain: a systematic review. Obesity (Silver Spring).

[29] Marshall NS, Glozier N, Grunstein RR (2008). Is sleep duration related to obesity? A critical review of the epidemiological evidence. Sleep Med Rev.

[30] Bjorvatn B, Sagen IM, Oyane N, Waage S, Fetveit A, Pallesen S (2007). The association between sleep duration, body mass index and metabolic measures in the Hordaland Health Study. J Sleep Res.

[31] Jean-Louis G, Williams NJ, Sarpong D, Pandey A, Youngstedt S, Zizi F (2014). Associations between inadequate sleep and obesity in the US adult population: analysis of the national health interview survey (1977–2009). BMC Public Health.

[32] Grandner MA, Chakravorty S, Perlis ML, Oliver L, Gurubhagavatula I (2014). Habitual sleep duration associated with self-reported and objectively determined cardiometabolic risk factors. Sleep Med.

[33] Theorell-Haglow J, Berglund L, Berne C, Lindberg E (2014). Both habitual short sleepers and long sleepers are at greater risk of obesity: a population-based 10-year follow-up in women. Sleep Med.

[34] Lucassen EA, Rother KI, Cizza G (2012). Interacting epidemics? Sleep curtailment, insulin resistance, and obesity. Ann N Y Acad Sci.

[35] Kripke DF, Simons RN, Garfinkel L, Hammond EC (1979). Short and long sleep and sleeping pills. Is increased mortality associated?. Arch Gen Psychiatry.

[36] Kripke DF, Langer RD, Elliott JA, Klauber MR, Rex KM (2011). Mortality related to actigraphic long and short sleep. Sleep Med.

[37] Dinges DF, Pack F, Williams K, Gillen KA, Powell JW, Ott GE (1997). Cumulative sleepiness, mood disturbance, and psychomotor vigilance performance decrements during a week of sleep restricted to 4–5 h per night. Sleep.

[38] Dinges DF, Douglas SD, Zaugg L, Campbell DE, McMann JM, Whitehouse WG (1994). Leukocytosis and natural killer cell function parallel neurobehavioral fatigue induced by 64 h of sleep deprivation. J Clin Invest.

[39] Spaeth AM, Dinges DF, Goel N (2013). Effects of Experimental Sleep Restriction on Weight Gain, Caloric Intake, and Meal Timing in Healthy Adults. Sleep.

[40] Van Dongen HP, Maislin G, Mullington JM, Dinges DF (2003). The cumulative cost of additional wakefulness: dose–response effects on neurobehavioral functions and sleep physiology from chronic sleep restriction and total sleep deprivation. Sleep.

[41] Rosekind MR, Gregory KB, Mallis MM, Brandt SL, Seal B, Lerner D (2010). The cost of poor sleep: workplace productivity loss and associated costs. J Occup Environ Med.

[42] Taheri S, Lin L, Austin D, Young T, Mignot E (2004). Short sleep duration is associated with reduced leptin, elevated ghrelin, and increased body mass index. PLoS Med.

[43] Patterson RE, Emond JA, Natarajan L, Wesseling-Perry K, Kolonel LN, Jardack P (2014). Short sleep duration is associated with higher energy intake and expenditure among African-American and non-Hispanic white adults. J Nutr.

[44] Spiegel K, Leproult R, Van Cauter E (1999). Impact of sleep debt on metabolic and endocrine function. Lancet.

[45] Spiegel R, Knudtson K, Leproult R, Tasali E, van cauter E (2005). Sleep loss: a novel rik factor for insulin resistance and Type II diabetes. J Appl Physiol.

[46] Jackson CL, Redline S, Kawachi I, Williams MA, Hu FB (2013). Racial disparities in short sleep duration by occupation and industry. Am J Epidemiol.

[47] Hale L, Do DP (2007). Racial differences in self-reports of sleep duration in a population-based study. Sleep.

[48] Tucker P, Smith L, Macdonald I, Folkard S (1998). The impact of early and late shift changeovers on sleep, health, and well-being in 8- and 12-h shift systems. J Occup Health Psychol.

[49] Pilcher JJ, Lambert BJ, Huffcutt AI (2000). Differential effects of permanent and rotating shifts on self-report sleep length: a meta-analytic review. Sleep.

[50] Hurtado DA, Sabbath EL, Ertel KA, Buxton OM, Berkman LF (2012). Racial disparities in job strain among American and immigrant long-term care workers. Int Nurs Rev.

[51] Murray-Bachmann R, Henry K, Ggrander MA, Ward K, Zizi F, Nunes J (2011). Social determinants of short sleep among black and white Americans. Sleep.

[52] Slopen N, Williams DR (2014). Discrimination, other psychosocial stressors, and self-reported sleep duration and difficulties. Sleep.

[53] Hicken MT, Lee H, Ailshire J, Burgard SA, Williams DR (2013). “Every shut eye, ain’t sleep”: The role of racism-related vigilance in racial/ethnic disparities in sleep difficulty. Race Soc Probl.

[54] Jackson CL, Hu FB, Redline S, Williams DR, Mattei J, Kawachi I (2014). Racial/ethnic disparities in short sleep duration by occupation: The contribution of immigrant status. Soc Sci Med.

[55] Mensah GA, Mokdad AH, Ford ES, Greenlund KJ, Croft JB (2005). State of disparities in cardiovascular health in the United States. Circulation.

[56] Redline S, Tishler P, Hans M, Tosteson T, Strohl K, Spry K (1997). Racial differences in sleep-disordered breathing in African-Americans and Caucasians. Am J Respir Crit Care Med.

[57] Ayas NT, White DP, Manson JE, Stampfer MJ, Speizer FE, Malhotra A (2003). A prospective study of sleep duration and coronary heart disease in women. Arch Intern Med.

[58] Ayas NT, White DP, Al Delaimy WK, Manson JE, Stampfer MJ, Speizer FE (2003). A prospective study of self-reported sleep duration and incident diabetes in women. Diabetes Care.

[59] Larkin EK, Patel SR, Goodloe RJ, Li Y, Zhu X, Gray-McGuire C (2010). A Candidate Gene Study of Obstructive Sleep Apnea in European-Americans and African-Americans. Am J Respir Crit Care Med.

[60] Cakirer B, Hans MG, Graham G, Aylor J, Tishler PV, Redline S (2001). The relationship between craniofacial morphology and obstructive sleep apnea in whites and in African-Americans. Am J Respir Crit Care Med.

[61] Buxbaum SG, Elston RC, Tishler PV, Redline S (2002). Genetics of the apnea hypopnea index in Caucasians and African Americans: I, Segregation analysis. Genet Epidemiol.

[62] QuickStats: Percentage of Adults Aged >18 Years* Who Reported an Average of <6 Hours of Sleep† per 24-Hour Period, by Sex and Age Group --- National Health Interview Survey, United States, 1985 and 2006. http://www.cdc.gov/mmwr/preview/mmwrhtml/mm5708a8.htm; Accessed 10-10-2012.

[63] Knutson KL, Van CE, Rathouz PJ, DeLeire T, Lauderdale DS (2010). Trends in the prevalence of short sleepers in the USA: 1975–2006. Sleep.

[64] Knight BG, McCallum TJ (1998). Heart rate reactivity and depression in African-American and white dementia caregivers: reporting bias or positve coping?. Aging Ment Health.

[65] Haley WE, Roth DL, Coleton MI, Ford GR, West CA, Collins RP (1996). Appraisal, coping, and social support as mediators of well-being in black and white family caregivers of patients with Alzheimer’s disease. J Consult Clin Psychol.

[66] Adenekan B, Pandey A, McKenzie S, Zizi F, Casimir GJ, Jean-Louis G (2013). Sleep in America: role of racial/ethnic differences. Sleep Med Rev.

[67] Jean-Louis G, Magai C, Cohen CI, Zizi F, von Gizycki H, DiPalma J (2001). Ethnic differences in reported sleep problems in older adults. Sleep.

[68] Jean-Louis G, Magai C, Consedine NS, Pierre-Louis J, Zizi F, Casimir GJ (2007). Insomnia symptoms and repressive coping in a sample of older Black and White women. BMC Womens Health.

[69] Leger D, Beck F, Richard JB, Sauvet F, Faraut B, Horne J (2014). The Risks of Sleeping “Too Much”. Survey of a National Representative Sample of 24671 Adults (INPES Health Barometer) Obesity and short sleep: unlikely bedfellows?. PLoS One.

[70] Ruiter ME, Decoster J, Jacobs L, Lichstein KL (2011). Normal sleep in African-Americans and Caucasian-Americans: A meta-analysis. Sleep Med.

[71] Reynolds AM, Bowles ER, Saxena A, Fayad R, Youngstedt SD (2014). Negative Effects of Time in Bed Extension: A Pilot Study. J Sleep Med Disord.

[72] Lauderdale DS, Knutson KL, Yan LL, Liu K, Rathouz PJ (2008). Self-reported and measured sleep duration: how similar are they?. Epidemiology.

[73] Youngstedt SD, Goff EE, Reynolds AM, Kripke DF, Irwin MR, Bootzin RR, Khan N, Jean-Louis G. Has adult sleep duration declined over the last 50+ years? Sleep Med Rev. 2015;28:65–81. 10.1016/j.smrv.2015.08.004 PMC4769964 26478985

[74] Jean-Louis G, Youngstedt D, Grandner M, Williams N, Sarpong D, Zizi F, Ogedegbe G, Unequal burden of sleep-related obesity among black and white Americans. Sleep Health, 1:169–176. 10.1016/j.sleh.2015.07.003 PMC4770938 26937487

